# Safety of Propofol versus Nonpropofol-Based Sedation in Children Undergoing Gastrointestinal Endoscopy: A Systematic Review and Meta-Analysis

**DOI:** 10.1155/2018/6501215

**Published:** 2018-07-30

**Authors:** Neeraj Narula, Sameer Masood, Samira Shojaee, Brandon McGuinness, Saama Sabeti, Arianne Buchan

**Affiliations:** ^1^Department of Medicine (Division of Gastroenterology) and Farncombe Family Digestive Health Research Institute, McMaster University, Hamilton, ON, Canada; ^2^Harvard TH Chan School of Public Health, Boston, MA, USA; ^3^Department of Medicine (Division of Pulmonary and Critical Care Medicine), Virginia Commonwealth University, Richmond, VA, USA; ^4^Department of Medicine, The Ottawa Hospital, Ottawa, ON, Canada

## Abstract

**Background:**

The majority of children who undergo gastrointestinal (GI) endoscopy require anesthesia or procedural sedation for comfort, cooperation, and procedure efficiency. The safety profile of propofol is not well established in children but has been studied in the literature.

**Objective:**

The aim of this study is to evaluate and compare the safety of propofol-only sedation for GI endoscopy procedures to other anesthetic regimes in the pediatric population.

**Methods:**

A search was conducted in the MEDLINE, Embase, and Cochrane Library databases. Randomized clinical trials and prospective cohorts were included in the study.

**Results:**

No significant difference was noted in total complications between the two cohorts with a pooled OR of 1.31 (95% CI: 0.57–3.04, chi^2^ = 0.053, *I* ^2^ = 54.31%). The pooled rate of complications in the studies was 23.4% for those receiving propofol only and 18.2% for those receiving other anesthetic regimens. Sensitivity analysis was performed removing a study with a very different control comparison compared to the rest of the studies included. Once excluded, there was minimal heterogeneity in the remaining studies and a significant difference in overall complications was detected, with more complications seen in the propofol-only group compared to the other anesthetic groups (OR 1.87, 95% CI 1.09–3.20).

**Conclusion:**

Significantly higher incidence of cardiorespiratory complications was noted in the propofol-only versus other anesthetic regimens in pediatric patients undergoing GI endoscopy in this meta-analysis. However, the overall quality of the evidence is very low.

**How to Apply This Knowledge for Routine Clinical Practice:**

Clinicians providing sedation to a pediatric population for GI endoscopy should consider there may be increased risks when using a propofol-only regimen, but further study is needed.

## 1. Introduction

Gastrointestinal (GI) endoscopic procedures are performed for diagnostic and therapeutic purposes in pediatric gastrointestinal diseases. Most children however do not tolerate these procedures without sedation or general anesthesia [1]. Proper sedation is particularly important for successful procedure completion, as well as preventing unpleasant experiences, which could complicate performing future procedures in children who will be frequently requiring them [2].

The goals of sedation are to ensure patient safety, provide analgesia and amnesia, control behavior during the procedure, enable successful completion of the procedure, and return the patient to pretreatment level of consciousness as fast as possible [3]. The most commonly used medication combinations for pediatric sedation include ketamine, propofol, midazolam, fentanyl, and pethidine [4]. Each of these drugs, however, can cause respiratory depression [5, 6].

The adverse respiratory profile of most sedatives creates the need for a sedative drug that can be used safely and efficiently with limited adverse effects during sedation in pediatric patients undergoing upper endoscopic procedures. Propofol (2,6-diisopropylphenol), an intravenously administered anesthetic released for use in 1989, has become popular as an agent for induction and maintenance of anesthesia [7]. The main advantages of propofol are rapid induction of anesthesia and smooth maintenance of sedation with good preservation of cardiovascular parameters [8–11].

Recovery from propofol is also remarkably rapid, occurring within 6 to 8 minutes and with fewer complications such as agitation, nausea, or vomiting, in the postprocedure period, which facilitates early discharge [12]. This favorable efficacy of propofol has made it a popular anesthetic agent for GI endoscopy. However, most experience with propofol relates to its use in adults [12, 13]. The safety of propofol in the pediatric age group has been documented during surgical, ophthalmologic, urologic, radiologic, and dental procedures [14–16]. However, data regarding its use for pediatric GI endoscopic procedures are limited and remain a matter of debate. In this meta-analysis, we present a review and meta-analysis of the existing literature evaluating the safety of a propofol-only sedation regimen compared to other sedation regimens during endoscopic procedures in the pediatric population.

## 2. Method Section

A search of Medline and Embase was performed using OVID for article retrieval and removal of duplicates. Using the same search terms, this search was also carried out in the Cochrane database of randomized controlled trials. Duplicates were removed manually by the reviewer for the Cochrane registry results. The following search terms were used:

1. Titles or abstracts containing endoscopy or esophagoscopy or aesophagoscopy or colonoscopy or sigmoidoscopy or endoscopic or enteroscopy or esophagogastroduodenoscopy or aesophagogastroduodenoscopy or gastroscopy or digestive system or gastrointestinal
2. Titles or abstracts containing propofol or diprivan or propoven
3. Titles containing anesthesia or anaesthesia or anesthetic or anaesthetic or sedat^∗^
4. 1 and 2 or 3
5. Filters for age 0–18 and publication year > 1980 which were applied appropriate to the respective database. For the Cochrane database, a childhood hedge which was applied:

Titles or abstracts containing child^∗^ or children or teen^∗^ or preteen^∗^ or pre-teen^∗^ or baby or babies or adolescent or youth or juvenile or toddler or pubescent or prepubescent or infant or newborn or pediatric or paediatric

To assess the sensitivity of the age filter, the childhood hedge was compared to the filter within Medline with no difference noted. The anesthesia term was restricted to a title search, due to a significant number of nonrelevant trials, returning with an abstract and title search. This search strategy was trialed against a list of previously identified trials, with none being excluded.

Titles and abstracts were screened independently by two investigators (Saama Sabeti, Brandon McGuinness). Inclusion criteria for progression to full-text review included the following: prospective cohort studies, randomized controlled trials (RCTs), pediatric population (<18 years old), GI endoscopy under sedation, and at least one propofol-only sedation arm. We decided to include studies where the control arm may have received propofol as well, but in addition to other anesthetic drugs, as the propofol dose used in this circumstance is often less than that used when relying on propofol only. Exclusion criteria included adult population, non-English, no propofol-only arm, no sedation comparisons, general anesthesia comparison, non-GI endoscopy procedure, not assessing outcomes of interest, and study designs other than prospective cohort studies or RCTs. The primary outcome of interest was total cardiac and respiratory complications. We also looked at secondary outcomes of cardiac and respiratory complications individually.

Once included studies had been determined, using a standardized data extraction form, two independent reviewers recorded data from the full text. For conference abstracts or articles where the full text was not available, data was collected from the abstract. The quality of the evidence was assessed for each included study. The Cochrane Risk of Bias Tool was used for randomized controlled trials, and the Newcastle-Ottawa scale was used for prospective cohort studies.

## 3. Statistical Analysis

Meta-analysis of aggregate patient data was conducted by combining the odds ratios of individual studies into a pooled odds ratio using a random effects model. Intention-to-treat data was extracted from all studies. We tested for heterogeneity using the chi-squared test and the *I* ^2^ test. The *I* ^2^ test describes the percentage of variability in effect estimates that is due to heterogeneity rather than chance, wherein an *I* ^2^ test greater than 50% suggests significant heterogeneity. A random effects model was used given the variation among study designs, as this provides a more conservative estimate than a fixed effects model. Metaregression was performed to analyze for an association between the quality of the study and the outcome of interest. This was specified a priori as a possible source of heterogeneity within our result pool, as prospective trials would be at a higher risk of confounding by indication. Another source of heterogeneity which was specified a priori was differences between studies in the sedation regimen used in the control group. For the assessment of publication bias, we performed funnel plots and calculated Egger's regression intercept for studies that report therapeutic response; a one-tailed *p* value < 0.05 was considered statistically significant. Analyses were performed with Comprehensive Meta-Analysis statistical software (version 2.2; Biostat, Englewood, NJ).

## 4. Results

**Figure 1:**
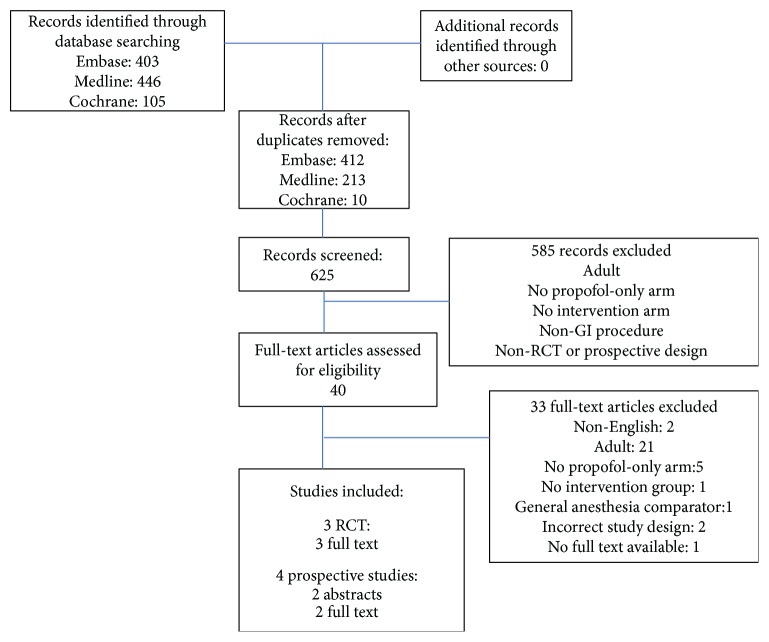
Prisma flow diagram. Retrieved studies and process of screening. Total included studies included 3 randomized controlled trials and 4 prospective cohort studies. All exclusion criteria were determined a priori.

In total, the search resulted in 625 titles and abstracts to be screened. The search process is summarized in a Prisma flow diagram (Figure 1). Forty studies progressed to a full-text review. After the full-text review, there were 2 studies excluded for being published in a non-English language, 21 studies excluded for involving an adult population, 5 studies excluded for not including a propofol-only arm, 1 study excluded for not having an intervention group, 1 study excluded for having a general anesthesia comparator as opposed to conscious sedation, and 2 studies excluded for an incorrect study design. The final list of 7 studies [17–23] selected to undergo data extraction included 3 RCTs for which full text was available and 4 prospective studies, 2 of which had full text available but 2 of which were only available in abstract form (full text exists but is not available online [17]; conference abstract only [23]).

Raw data is summarized in Table 1. This was subsequently condensed into combined cardiovascular and respiratory complications (Table 2). Adverse cardiovascular outcomes were defined as hypotension or bradycardia. Adverse respiratory outcomes were defined as apneic episodes, depressed respiratory rate, or hypoxia. A combined total cardiorespiratory adverse event outcome was assigned for each study. One study [22] included only data measured as the median integrated pulmonary index (IPI), a combined measure of both cardiac and respiratory parameters. This however was not directly correlated with harm in the study, and no harm data was reported. Therefore, for the purpose of analysis, it was excluded. The median IPI however was lower (1 Q1:1 Q3:7) than that for the propofol + midazolam (7 Q1:6 Q3:8) or propofol + midazolam + fentanyl group (7 Q1:6 Q3:8). These results are consistent with the subsequent analysis. All remaining studies included data regarding respiratory complications. Four of the included studies reported the number of cardiovascular complications. Nausea and emesis as well as recovery time were not included in the final analysis as it was only reported in one and three studies, respectively.

### 4.1. Total Cardiovascular and Respiratory Complications

**Figure 2:**
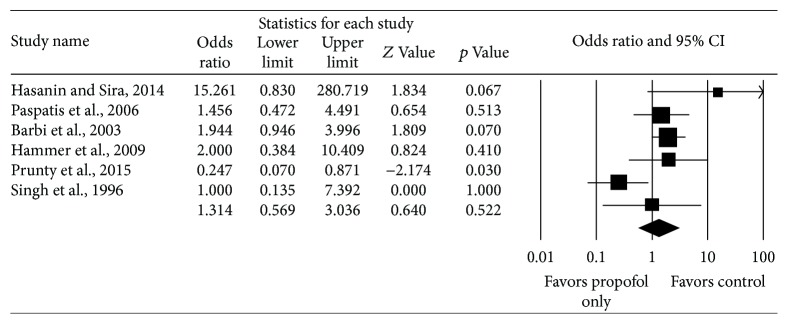
Comparison of total complications in children receiving propofol only versus other anesthetic regimens.

Six studies reported total complications from endoscopy and included 273 subjects who received propofol only and 319 subjects received other anesthetic regimens. There was no significant difference in total complications between the two cohorts, as the pooled OR for total complications among the six studies was 1.31 (95% CI: 0.57–3.04, Cochrane's *Q* statistic *p* value = 0.053, *I* ^2^ = 54.31%) (Figure 2). The pooled rate of complications in the studies is 23.4% for those receiving propofol only and 18.2% for those receiving other anesthetic regimens.

### 4.2. Respiratory Complications

**Figure 3:**
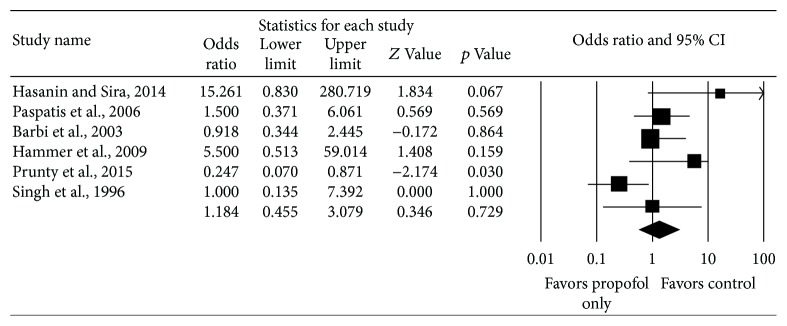
Comparison of respiratory complications in children receiving propofol versus under anesthetic regimens.

Six studies reported respiratory complications from endoscopy and included 273 subjects who received propofol only and 319 subjects received other anesthetic regimens. There was no significant difference in respiratory complications between the two cohorts, with a pooled OR for respiratory complications among the six studies of 1.18 (95% CI: 0.46–3.08, Cochrane's *Q* statistic *p* value = 0.07, *I* ^2^ = 52%) (Figure 3). The pooled rate of respiratory complications in the studies is 11.0% for those receiving propofol only and 10.7% for those receiving other anesthetic regimens.

### 4.3. Cardiovascular Complications

**Figure 4:**
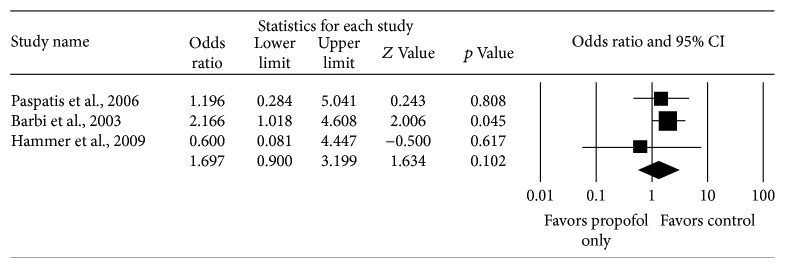
Comparison of cardiac complications in children receiving propofol versus under anesthetic regimens.

Four studies reported cardiovascular complications from endoscopy and included 140 subjects who received propofol only and 140 subjects received other anesthetic regimens. There was no significant difference in cardiovascular complications between the two cohorts, with a pooled OR for cardiovascular complications among the four studies of 1.70 (95% CI: 0.90–3.20, Cochrane's *Q* statistic *p* value = 0.44, *I* ^2^ = 0%) (Figure 4). The pooled rate of cardiovascular complications in the studies is 24.3% for those receiving propofol only and 17.1% for those receiving other anesthetic regimens.

### 4.4. Sensitivity Analysis and Publication Bias

**Figure 5:**
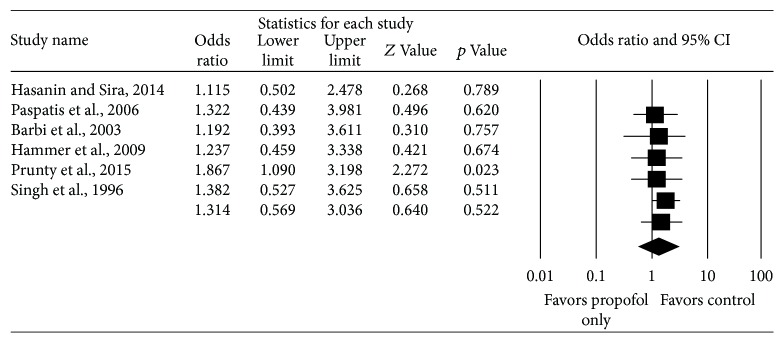
Sensitivity analysis with one study removed.

**Figure 6:**
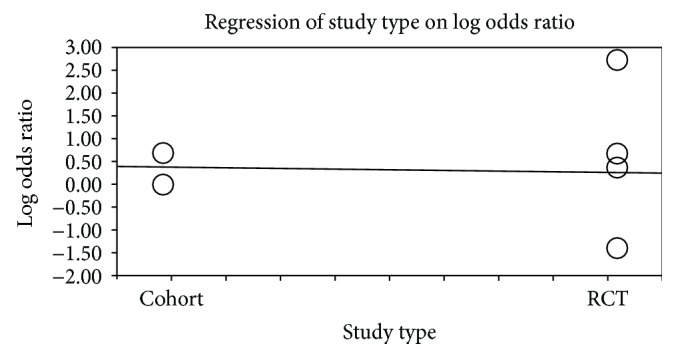
Plot of metaregression.

**Figure 7:**
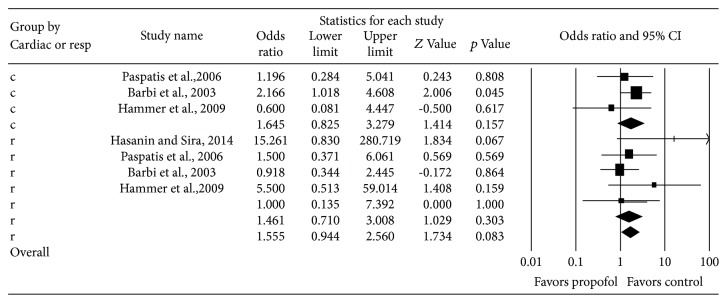
Subgroup analysis.

Sensitivity analyses were performed using the one-study removed technique (Figure 5). A significant difference in overall complications was detected once the Prunty et al. study was removed, with less complications seen in the other anesthetic regimens compared to propofol only (OR 1.87, 95% CI 1.09–3.20). The study by Prunty et al. used a four-drug combination in the control arm, including propofol, and it is possible that they observed an excess of respiratory complications due to this. Inclusion of this study in the overall meta-analysis led to more complication events occurring in the control arm and as a result led to no significant difference in total complications between those receiving propofol only and other anesthetic regimens in the overall meta-analysis. We felt this study was inherently different from the remaining studies included in the meta-analysis given the excessive number of drugs used on the control arm. Once this study was excluded, there was minimal heterogeneity between the remaining studies detected (Cochrane's *Q* statistic *p* value = 0.63, *I* ^2^ = 0%). Metaregression was performed to adjust for study type (RCT compared to cohort studies). No significant difference was seen between RCTs and cohort studies (*p* = 0.87) (Figure 6). We conducted a subgroup analysis of cardiovascular versus respiratory complications to determine if one subgroup had a more significant effect seen in total complications (excluding the study by Prunty et al. given its contribution to increasing heterogeneity). We did not detect any significant difference between cardiovascular and respiratory complications (*p* = 0.08) (Figure 7).

**Figure 8:**
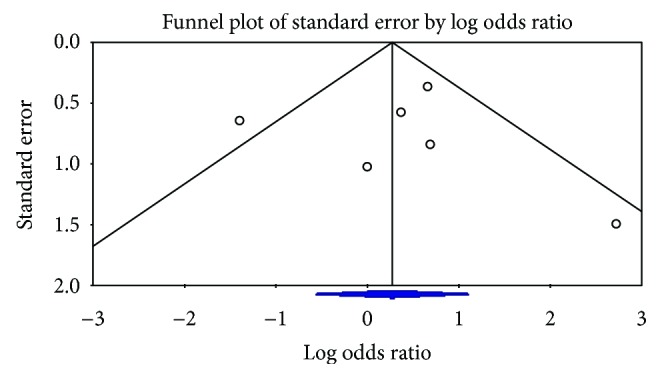
Funnel plot for publication bias.

Figure 8 is a funnel plot for studies that report on total complications. Symmetrical distribution of the studies on the plot suggests no publication bias. Egger's test similarly showed no publication bias (*p* value = 0.46) but may have limited ability to detect publication bias given the small number of studies included.

Bias assessment for each study is provided in supplementary materials (available here). The overall quality of the included studies is summarized in Table 3 and Supplementary Figure S1. Randomized controlled trials frequently did not specify the method of allocation or blinding. Within the observational trials, none demonstrated good comparability between the trial arms. Therefore, there was a high risk of confounding by indication within the included studies. According to the GRADE system for assessing quality, evidence from randomized controlled trials begins with a “high” rating. We downgraded the rating because of the risk of bias in the studies, given that patients and clinicians were not blinded, as well as impreciseness of the treatment effect and inconsistency in results. The overall GRADE rating applied to the randomized controlled trials was “low.” Evidence from observational evidence begins with a “low” rating. We downgraded the rating to “very low” due to the risk of bias in some of the observational studies, mainly due to inclusion of abstracts where the risk of bias was largely unclear, as well as imprecision of the treatment effect due to a small size. Details are available in Table 3.

## 5. Discussion

Our results show that there was a statistically significant difference in combined cardiorespiratory complications between propofol-only and other sedation regimens in pediatric patients undergoing GI endoscopy, when excluding the study by Prunty et al. which we felt used an inappropriate control group for the purposes of our safety analysis. There was no difference in the subgroup analysis when complications were divided into cardiovascular and respiratory.

Our study focused on the safety of propofol compared to other sedation regimens in pediatric patients, specifically in relation to respiratory or cardiovascular complications. To our knowledge, there has not been a previous meta-analysis addressing this topic. A previous systematic review examined 10 studies (6 RCTs, 4 non-RCTs) looking at procedural sedation for GI endoscopy in children [24]. This systematic review concluded that propofol-based sedation was a safe option with low rates of major respiratory complications with the use of propofol and did not find a significant difference in adverse events compared to other sedative regimens [24]. However, since this study did not perform a meta-analysis and its conclusions were based on a review of heterogeneous studies, the overall safety was difficult to quantify.

Previous studies in the adult population have shown that propofol is an effective and safe agent to use for sedation in GI endoscopy procedures [25]. Most studies in children have centered around effectiveness, including which physician specialty is best suited to administer the sedation [26]. The efficacy of propofol as a sedative agent for endoscopy is well established, largely in part due to its rapid onset, short-acting nature, low cost, and decades of experience using it in adults. However, safety data on propofol in children undergoing endoscopy is lacking and concern has been raised due to its potential to induce respiratory depression and cardiovascular instability [26]. A survey of pediatric gastroenterologists found that there is a wide variation in practice with respect to sedation for GI endoscopy in pediatric patients, and this included experience with propofol [27].

There have been several meta-analyses designed to answer this question in the adult population. Most recently, in 2017, Wadhwa et al. published a meta-analysis that compared propofol to other anesthetics for GI endoscopy in the adult population [25]. The authors concluded that based on a meta-analysis of 27 original studies, there was no significant difference in cardiopulmonary adverse events between these two groups [25]. Notably, our meta-analysis showed that results differed in a pediatric population.

Our study has several strengths. It is the first meta-analysis done specifically on the pediatric population which attempts to answer an important question. We strictly included prospective studies, which minimized bias seen in previous observational studies. The focus of the analysis on overall cardiorespiratory complications is an important distinction from other complications due to their direct relationship to mortality. Furthermore, the meta-analysis also provided insights into the current state of evidence, including the need for standardized reporting of adverse outcomes during procedural sedation and the lack of publication bias being a significant factor in the study findings. Stratifying our studies based on the study design did not change our overall findings, which adds credibility and consistency to our overall findings as study design bias did not affect the results.

Several limitations were identified in this meta-analysis. First, very few studies exist that address the study question. Only a total of 6 studies were included and generated a pooled sample size of only 592 patients. The individual studies all had small sample sizes, the largest of which was Prunty et al. which included 212 patients [23]. As compared to a recent meta-analysis done in the adult population which included 27 studies totaling 2518 patients [25], we believe that the lack of studies and small sample size were in part due to the generally lower number of research studies done in pediatric populations which was the focus of our question.

Second, the quality of the studies identified in this meta-analysis was assessed using the GRADE criteria. We concluded that the quality of the RCTs and the prospective studies included ranked very low. The main reasons for the poor quality included unclear randomization protocols, lack of blinding, and lack of concealment of allocation.

Third, the other major weakness of this meta-analysis was heterogeneity. To address this issue, we performed the “one-study” removed analysis (removed Prunty et al.) and found that it significantly reduced the heterogeneity. A priori, we had a concern that the variation in comparison arm sedation regimens between studies was a large source for heterogeneity. Prunty et al. included four different sedative agents (propofol + glycopyrrolate + fentanyl + midazolam) in the comparison arm [23]. It is possible that combining multiple sedatives may in fact increase the risk of adverse events through an additive or synergistic mechanism. This also makes the study different from the rest, as the other comparison arms consist of only one or two sedative agents. Therefore, we opted to present results of the “one-study” removed analysis excluding Prunty et al. With this study excluded, there was a statistically significant increase in combined cardiovascular and respiratory adverse events using propofol only. It is important to note however that while *I* ^2^ became 0% with this change, there are still differences between the studies and wide confidence intervals. We may not have the power to detect differences within studies. For example, we were interested in cardiovascular complications including hypotension. Only 4 studies [18–21] included hypotension as an adverse effect, and even between these studies, the outcome measure was defined differently. Barbi et al. defined hypotension as a drop in systolic blood pressure (SBP) over 20 mmHg [18], whereas Paspatis et al. defined it as a drop in SBP over 10 mmHg [19]. Given the inconsistencies in outcomes between the studies, the different outcome measures reported were grouped to create broader categories consisting of respiratory complications, cardiovascular complications, and combined to report total complications. Similarly, procedural details differed between studies; one notable example was by the type of endoscopy. The majority of studies included upper GI endoscopy, but one included lower GI endoscopy as well [21]. These results were not presented separately; therefore, we could not perform metaregression based on the procedure type. As well, patient details including the reason for undergoing endoscopy and comorbid conditions were not clearly reported and may have influenced complication rates. Finally, the sedation regimen varied within the comparison arm between studies. Of the 5 studies included in the “one-study” removed analysis, 4 studies contained a propofol + additional sedative comparison arm consisting of propofol + dexmedetomidine [20], midazolam + propofol [19], ketamine + propofol [18], and diazemul or pentazocine + propofol [17]. Only one study did not contain propofol in the comparison arm, instead of using dexmedetomidine as the comparator [21]. It is therefore important to interpret our results in the context of these studies. We are unable to draw a conclusion regarding an ideal sedative regime; however, there may be an increase in total cardiorespiratory complications when using propofol only.

## 6. Conclusion

Our meta-analysis found a significant difference in combined cardiorespiratory adverse events when propofol-only sedation was used for pediatric patients undergoing endoscopy compared to other sedation regimens. The odds of combined adverse events was higher in pediatric patients who received propofol only for sedation during GI endoscopy (OR 1.87, 95% CI 1.09–3.20). This is not in keeping with prior studies or with a recent meta-analysis done in the adult population. This meta-analysis was designed to specifically examine cardiorespiratory adverse events. It did not address the efficacy of propofol compared to other sedation regimens. We would recommend that the findings from this meta-analysis be taken in conjunction with data on efficacy in order to best guide clinical practice.

There were multiple limitations encountered in this study, including very low-quality evidence; therefore, we believe further higher quality evidence is needed. We recommend a well-designed, large study focusing on well-defined safety outcome measures to convincingly answer our study question. A large multicenter prospective cohort study where patients undergoing different sedation regimens were observed and recorded would be the most feasible and practical way to definitively answer this question. Until then, health care providers providing sedation to children should consider the possibility of increased risk in total complications and weigh this against possible benefits in using propofol only.

## Figures and Tables

**Table 1:** Detail summarizing the 3 randomized controlled trials and 4 prospective studies included in the meta-analysis.

Study	Age	Procedure	Treatment arms	*N*	Dose	Adverse events (*n*)				
						Bradycardia	Hypotension	Desaturation	Respiratory depression	Total airway
Singh et al., 1996 [17] Prospective cohort (abstract) *N* = 100	4–15	Endoscopy, colonoscopy	Propofol	50	3–3.5 mg/kg				2 (apnea)	
			Propofol + diazemul or pentazocine	50	Propofol 2.5–3 mg/kg, diazemul 2.5 mg, pentazocine 5 mg				2 (apnea)	

Barbi et al., 2003^∗^ [18] RCT (full text) *N* = 122	0.5–17	Upper GI	Propofol	60	n/a		27	8		
			Propofol + ketamine	62	n/a		17	9		

Paspatis et al., 2006^∗∗^ [19] RCT (full text) *N* = 54	3–17	Upper GI	Propofol	28	0.5 mg/kg repeat doses	4	1	6		
			Propofol + midazolam premed (PO)	26	Propofol: 0.5 mg/kg repeat doses (max 20 mg)	4	0	4		

Hammer et al., 2009 [20] Prospective cohort (full text) *N* = 24	3–10	EGD	Propofol	12	Avg: 2.8–4 mcg/ml	0	2		4	
			Propofol + dexmedetomidine	12	Dex: bolus (10 min) 1 mcg/kg; maintenance: 0.5 mcg/kg/hr; propofol: avg 2.8–4 mcg/ml	3	0		1	

Hasanin and Sira 2014 [21] RCT (full text) *N* = 80	1–14	Upper and lower endoscopy	Propofol	40	Bolus: 2 mg/kg; maintenance:100 mcg/kg/min	0	0	6	0	
			Dexmedetomidine	40	Bolus: 2.5 mcg/kg; maintenance: 2 mg/kg/hr	0	0	0	0	

Garah et al., 2015 [22] Prospective cohort (full text) *N* = 109	1–18	Upper endoscopy, colonoscopy, both	Propofol	5						IPI 1 (IQR 6)
			Propofol + midazolam	89						IPI 7 (IQR 2)
			Propofol + midazolam fentanyl	15						IPI 7 (IQR 2)

Prunty et al., 2015 [23] Prospective cohort (abstract) *N* = 212	n/a	EGD	Propofol	83				3	0	33
			Propofol + glycopyrrolate + fentanyl + midazolam	129				15	2	17

EGD: esophagogastroduodenoscopy. ^∗^Definition of hypotension: mean arterial pressure (MAP) decrease of >20 mm hg; definition of desaturation: SpO2 < 92%. ^∗∗^Definition of bradycardia: 20% decrease in heart rate; definition of hypotension: MAP decrease > 10 mm hg; definition of desaturation: hypoxemia < 92% for 10 seconds.

**Table 2:** Condensed data set. Data was collapsed into the following categories from the raw data, Table 1, using the following criteria; cardiovascular depression included hypotension or bradycardia; respiratory depression included hypoxia, decreased respiratory rate, or apnea.

Study	Group	Cardiovascular depression (*n*)	Respiratory depression (*n*)
Singh et al., 1996	Propofol (*n* = 50)		2
	Propofol + diazemul or pentazocine (*n* = 50)		2

Barbi et al., 2003	Propofol (*n* = 60)	27	8
	Propofol + ketamine (*n* = 62)	17	9

Paspatis et al., 2006	Propofol (*n* = 28)	5	6
	Propofol + midazolam (*n* = 26)	4	4

Hammer et al., 2009	Propofol (*n* = 12)	2	4
	Propofol + dexmedetomidine (*n* = 12)	3	1

Hasanin and Sira, 2014	Propofol (*n* = 40)	0	6
	Dexmedetomidine (*n* = 40)	0	0

Prunty et al., 2015	Propofol (*n* = 83)		3
	Propofol + glycopyrrolate + fentanyl + midazolam (*n* = 129)		17

**Table 3:** Overall quality assessment, on basis of the Grading of Recommendations Assessment, Development, and Evaluation (GRADE) criteria.

Study	Starting level of evidence	Reasons for decreasing the level of evidence					Reasons to increase level of evidence (strong association, plausible confounding, and bias adjustment)	Final level of evidence
		Risk of bias	Inconsistency	Indirectness	Imprecision	Publication bias		
Randomized trials—3	High	↓	↓	↔	↓	↔	↔	Very Low
Observational trials—4	Low	↓	↔	↔	↓	↔	↔	Very low

## Supplementary Materials

Supplementary Materials Risk of bias assessment.
