# Supplementary material for: Safety of Propofol versus Nonpropofol-Based Sedation in Children Undergoing Gastrointestinal Endoscopy: A Systematic Review and Meta-Analysis
Source: Gastroenterol Res Pract. 2018 Jul 30;2018:6501215. doi: 10.1155/2018/6501215 (PMC6126059; doi:10.1155/2018/6501215)
Supplement: Supplementary Materials — Risk of bias assessment. [file 6501215.f1.pdf]

Supplemental Material

Risk of bias assessment

Study: Prunty M, 2016

Selection

1. Representation of the exposed cohort (d)
2. Selection of the non-exposed cohort (a)*
3. Ascertainment of exposure (a)*
4. Demonstration that outcomes of interest was not present at start of study (a)*

Comparability

1. Comparability of cohorts on the basis of the design or analysis: No description of controlled variables

Outcome

1. Assessment of Outcome (d)
2. Was follow-up long enough for outcomes to occur (a)*
3. Adequacy of follow up of cohorts (a)*

Total Newcastle-Ottawa score: 5

Study: Singh et al, 1996

Selection

1. Representation of the exposed cohort (d)
2. Selection of the non-exposed cohort (a)*
3. Ascertainment of exposure (a)*
4. Demonstration that outcomes of interest were not present at start of study (a)*

Comparability

1. Comparability of cohorts on basis of design or analysis: No description

Outcome

1. Assessment of outcome (d)*
2. Was follow-up long enough for outcomes to occur (a)*
3. Adequacy of follow-up of cohorts

Total Newcastle-Ottawa score: 5

Study: Hammer G, 2009

Selection

1. Representation of the exposed cohort (b) *
2. Selection of the non-exposed cohort (a)*
3. Ascertainment of exposure (a)*
4. Demonstration that outcomes of interest was not present at start of study (a) *

Comparability

1. Comparability of cohorts on the basis of the design or analysis: No discussion of controlling for other variables.

Outcome

1. Assessment of Outcome (b)*
2. Was follow-up long enough` for outcomes to occur (a)*
3. Adequacy of follow up of cohorts (a)*

Total Newcastle-Ottawa score: 7

Study: Garah, 2015

Selection

5. Representation of the exposed cohort (a) *
6. Selection of the non-exposed cohort (a)*

7. Ascertainment of exposure (a)*
8. Demonstration that outcomes of interest was not present at start of study (a)*

Comparability

2. Comparability of cohorts on the basis of the design or analysis: Did not control for other variables.

Outcome

4. Assessment of Outcome (b)*
5. Was follow-up long enough` for outcomes to occur (a)*
6. Adequacy of follow up of cohorts (a)*

Total Newcastle-Ottawa score: 7

Hasanin. A, 2014

**Domain**	**Risk of Bias**	**Comments**
Sequence generation	Unclear	No mention of method of randomization.
Allocation concealment	Unclear	No mention of method of allocation process.
Blinding of participants	Unclear	No mention of blinding of patient.

Personnel and outcome assessors	High	No independent blinded assessor.
Incomplete outcome data	Low	Occurred in outpatient setting. No long term follow-up.
Selective outcome reporting	Low	Reported all outcomes.
Other sources of bias	Low	No other noted sources of bias

Barbi et al. 2003

**Domain**	**Risk of Bias**	**Comments**
Selection bias/<br>Sequence generation	Low	Computer generated randomization
Allocation concealment	Unclear	Does not specifically address concealment, although blinding implies
Blinding of participants, personnel	Low	Patients, doctor in charge of sedation and RN in charge of data collection were all blinded

Blinding of outcome assessors	Low	RN collecting the data was unaware of drug administered
Incomplete outcome data	Unclear	Not specifically addressed<br><br>122 patients were randomized, but study states “total of 112 gastroscopies were successfully performed”. Outcome table still reports N that toal 122. Unclear if data was collected on all 122 or just the 112 successful scopes.
Selective outcome reporting	Low	Primary and secondary outcomes have all been reported
Other sources of bias	Unclear	? misclassification bias - the discomfort scale was based on observed behaviours - validity of this tool is unknown

Paspatis et al. 2006

**Domain**	**Risk of Bias**	**Comments**
Selection bias/<br>Sequence generation	Low	Table of random numbers
Allocation concealment	Unclear	Does not address concealment

Blinding of participants, personnel	High	Not blinded for the endoscopist or the anesthesiologist. Pediatrician was blinded.
Blinding of outcome assessors	Unclear	The blinded pediatrician appeared to assess the efficacy, it does not address who assessed safety outcomes. Patients had continuous vital sign monitoring and an RN who was observing the patient - did not state of the RN was blinded.
Incomplete outcome data	Low	Appears that efficacy and safety data was collected on all patients (26 +28).<br><br>Discomfort scale was only collected on children over 6 years - this was clearly stated (20 + 21)
Selective outcome reporting	Low	It appears that all outcomes have been reported
Other sources of bias	Low	No obvious others

Summary of Bias:

Table A1: Newcastle-Ottawa assessment of non-randomized trials for all prospective cohort studies included.

Study	Selection	Comparability	Outcome
Singh et. al	***		**
Prunty et al.	***		**
Hammer et al.	****		***
Garah et al.	****		***

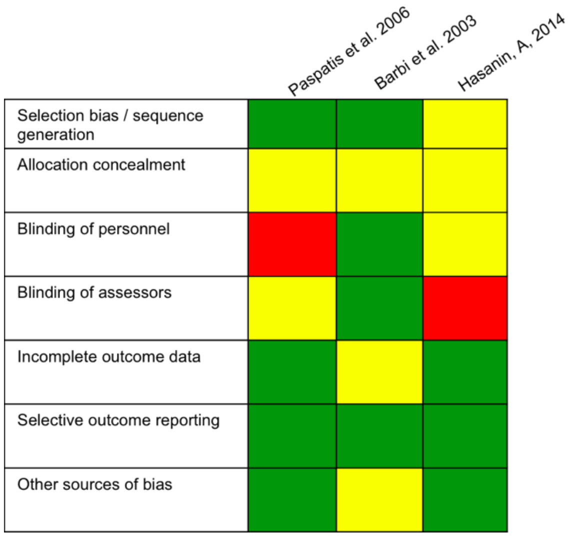

Figure S1: Assessment of randomized controlled trials using the Cochrane Risk of Bias Tool. Red indicates high risk of bias, yellow indeterminate risk of bias, green low risk of bias.
